# Transcriptomic and metabolomic analyses reveal the spatial role of carnitine metabolism in the progression of hepatitis B virus cirrhosis to hepatocellular carcinoma

**DOI:** 10.3389/fmicb.2024.1461456

**Published:** 2024-12-13

**Authors:** Pengxiang Gao, Qiuping Liu, Ziye Luo, Wenjun Pu

**Affiliations:** ^1^College of Life Sciences and Oceanography, Shenzhen University, Shenzhen, Guangdong, China; ^2^Clinical Medical Research Center, The Second Clinical Medical College, Jinan University (Shenzhen People's Hospital), Shenzhen, Guangdong, China; ^3^Medical Imaging Key Laboratory of Sichuan Province, North Sichuan Medical College, Nanchong, Sichuan, China

**Keywords:** hepatitis B virus, liver cirrhosis, hepatocellular carcinoma, transcriptomic, metabolomic, mass spectrometry imaging, carnitine metabolism

## Abstract

**Introduction:**

Liver cirrhosis (LC) and hepatocellular carcinoma (HCC) resulting from chronic hepatitis B virus (HBV) infection are major health concerns. Identifying critical biomarkers and molecular targets is needed for early diagnosis, prognosis, and therapy of these diseases.

**Methods:**

In this study, we explored the gene expression and metabolism in the liver tissues of LC, HCC, and healthy controls, to analyse and identify potential biomarkers of disease progression. Mass spectrometry imaging was used to evaluate the spatial distribution of key metabolites.

**Results and discussion:**

The results revealed significant changes in gene expression and metabolic pathways along with disease progression. The upregulated genes were associated with extracellular matrix remodeling and cancer pathways, including LAMC1-3, COL9A2, COL1A1, MYL9, MYH11, and KAT2A. The downregulated genes were linked to immune response and fatty acid metabolism. Metabolomic analysis showed major changes in lipid and choline metabolism. Consistent changes in the expression of specific genes and metabolites were correlated with clinical data. Notably, metabolites such as L-acetylcarnitine, histamine, and 4-trimethylammoniobutanoic acid demonstrated high accuracy (AUC > 0.85) in distinguishing between healthy, LC, and HCC groups. This study identifies key gene and metabolite changes in HBV related LC and HCC, highlighting critical pathways involved in disease progression. Biomarkers like L-acetylcarnitine and KAT2A show promise for early diagnosis and prognosis, potentially improving outcomes for hepatitis liver disease patients.

## 1 Introduction

Liver diseases, particularly liver cirrhosis (LC) and hepatocellular carcinoma (HCC), represent significant global health challenges, with increasing morbidity and mortality rates (Nartey et al., 2022). These conditions are often the result of chronic liver damage due to various etiologies, including viral infections, excessive alcohol consumption, and metabolic disorders (Wu X. N. et al., 2024). Among these, hepatitis B virus (HBV) infection is a predominant cause, especially in regions with high HBV prevalence. According to the WHO, nearly 300 million people worldwide are affected by HBV infection, which lead to severe liver complications (Hsu et al., 2023). Liver cirrhosis is characterized by widespread fibrosis, nodular regeneration, and the formation of abnormal liver architecture, known as pseudolobules (Pinzani et al., 2011). The progression from chronic hepatitis to cirrhosis and ultimately to HCC is marked by significant alterations in liver tissue architecture and cellular processes, including proliferation, apoptosis, angiogenesis, and the immune response (Llovet et al., 2021). Understanding the molecular mechanisms underlying this progression is crucial for identifying potential biomarkers and therapeutic targets to improve early diagnosis and treatment outcomes.

Previous studies have highlighted the role of specific gene expression changes and metabolic reprogramming in the progression of liver diseases. Studies have identified distinct metabolic signatures associated with this transition (Fan et al., 2021). One notable change is the dysregulation of amino acid metabolism, with increased levels of serine, glycine, and creatine observed in HCC compared to liver cirrhosis (Xie et al., 2022). Additionally, elevated levels of cystathionine and linoleic acid have been reported in HCC relative to cirrhotic liver tissues (Cai et al., 2020). These alterations are linked to the differential expression of enzymes such as AGXT2, DAO, CTH, BPGM, CBS, PSPH, and ACOT7, which play roles in amino acid metabolism (Cai et al., 2020). Lipid metabolism also undergoes substantial changes during the transition from cirrhosis to HCC, with dysregulation of glycerolipid, glycerophospholipid, and fatty acid metabolism pathways (He et al., 2022; Pu et al., 2023), aberrant activation of signaling pathways such as the Wnt/β-catenin and Hedgehog, and alterations in the extracellular matrix (ECM) have been implicated in the pathogenesis of LC and HCC (Gajos-Michniewicz and Czyz, 2024). The upregulation of enzymes involved in fatty acid biosynthesis and lipogenesis, such as FASN and ACC, has been observed in HBV-associated HCC (Cheng et al., 2024). Furthermore, alterations in glycan biosynthesis and metabolism pathways contribute to the metabolic reprogramming associated with hepatocarcinogenesis (Cheng et al., 2024). These metabolic shifts are closely intertwined with the dysregulation of key signaling pathways, including PI3K/Akt, mTOR, and HIF-1α, which play crucial roles in regulating cellular metabolism, proliferation, and survival (Hoxhaj and Manning, 2020). However, there is still a need for a more integrated analysis that combines transcriptomic and metabolomic data to elucidate the interplay between genetic and metabolic changes during disease progression.

Recent advances in high-throughput technologies, such as transcriptomics and metabolomics, have provided comprehensive insights into the molecular changes associated with liver diseases (Liang and Song, 2023). Transcriptomic analysis allows for the examination of gene expression profiles, revealing key regulatory pathways and gene networks involved in disease progression. Metabolomics, on the other hand, provides a detailed snapshot of metabolic alterations, reflecting the functional state of cells and tissues. However, traditional metabolomics studies mainly provide bulk information of metabolites in specific samples, which often lack spatial characteristics within organs as diseases progression. Understanding the spatial distribution of metabolites and the underlying signaling pathways is essential for elucidating the heterogeneity of HBV-related liver diseases and for constructing a comprehensive spatial metabolic network within the liver.

The metabolic reprogramming observed during the transition from HBV-induced liver cirrhosis to HCC provides potential biomarkers for early detection and therapeutic targets. In this context, the integration of transcriptomic and metabolomic data offers a synergistic approach to comprehensively characterize the molecular changes occurring during the transition from HBV-induced liver cirrhosis to HCC. By correlating gene expression patterns with metabolic alterations, researchers can identify potential biomarkers for early detection and targets for therapeutic intervention, ultimately improving clinical outcomes for patients with chronic HBV infection. In this study, we aimed to investigate the molecular and metabolite alterations using a combined transcriptomic and untargeted metabolomic approach. By analyzing liver tissue samples from the healthy control, liver cirrhosis and hepatocellular carcinoma groups, we sought to identify key genes and metabolic pathways associated with disease progression. In addition, mass spectrometry imaging was further applied to elucidate the spatial distribution of metabolites during disease progression. Moreover, the predictive value of key metabolites and genes was also evaluated to uncover the potential biomarkers role for early detection, and to explore new therapeutic targets.

## 2 Materials and methods

### 2.1 Sample collection and preparation

**Figure 1:**
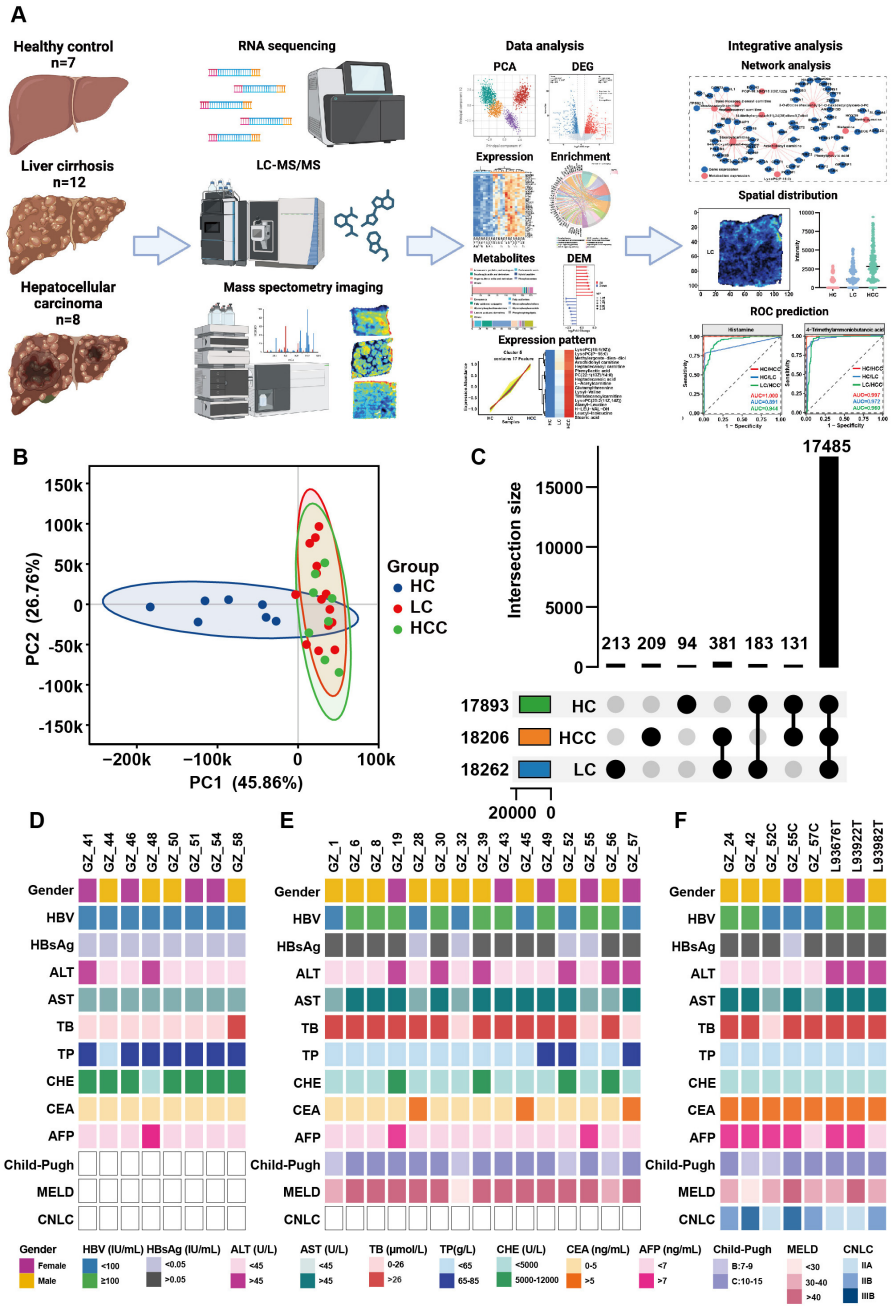
Study workflow and the clinical data of patients. **(A)** Overview of the study workflow. **(B)** PCA results for healthy controls (HCs), patients with HBV related liver cirrhosis (LC) and hepatocellular carcinoma (HCC). **(C)** Overlap of genes among the three groups. **(D-F)** Clinical data for the HC **(D)**, LC **(E)**, and HCC groups **(F)**.

Liver tissues from 15 patients with HBV related liver cirrhosis (LC), eight patients with HBV related hepatocellular carcinoma (HCC), and eight healthy controls (HC) were collected postsurgery at Shenzhen People's Hospital from January 2022 to July 2023. The study complied with the ethical guidelines of the Declaration of Helsinki and received ethical approval from the ethics committees of Shenzhen People's Hospital (LL-KY-2021723), and informed consent was obtained from all participants. The clinical data for all participants are presented in Figure 1. All patients tested positive for HBV infection, which was confirmed by detecting the HBV copy number and Hepatitis B surface antigen (HBsAg). For mass spectrometry imaging, the samples were embedded in OCT and sectioned into 10 μm thick slides using a Leica CM1950 cryostat microtome. At least three slides designated for hematoxylin and eosin (H&E) staining, positive ionization mode, and negative ionization mode of MSI were generated for each sample. For LC-MS/MS, samples were homogenized and extracted using an isotope-labeled internal standard mixture extraction solution (acetonitrile: methanol = 1:1).

### 2.2 AFAI-MSI

Mass spectrometry imaging (MSI) assays were performed using a custom-made AFAI ion source coupled with a Q-Exactive mass spectrometer (Thermo Fisher Scientific). The spray solvent mixed with acetonitrile and water (8:2, v/v) and additional added 0.1% formic acid at a flow rate of 5 μL/min. Nitrogen was used as the spray gas at a rate of 45 L/min, and the capillary temperature was set at 350°C. The MSI was performed by scanning the tissue slides at 200 and 100 μm in the x-direction and y-direction, respectively.

### 2.3 Untargeted metabolomic study

The LC-MS/MS analysis was executed using a Vanquish UHPLC system (Thermo Fisher Scientific), coupled to an Orbitrap Exploris 120 mass spectrometer (Thermo Fisher Scientific). Chromatography was performed in gradient mode and solvent A consisted of 25 mmol/L ammonium acetate and 25 mmol/L ammonia in water, while solvent B was acetonitrile. The injection volume was 2 μL and the flow rate was set at 0.5 mL/min. The column temperature was set at 30°C and the auto-sampler was maintained at 4°C. The initial gradient was 95% solvent B for 0.5 min, then decreased from 95 to 65% over 0.5–7 min, further decreased to 40% over 7–8 min, held at 40% B for 8–9 min, and reverted back to 95% B over 9–12 min.

The MS/MS spectra were managed with Xcalibur 4.4 acquisition software (Thermo Fisher Scientific). The sheath gas and auxiliary gas flow rates were at 50 Arb and 15 Arb, respectively. The capillary temperature was set at 320°C. The full MS resolution was at 60,000, the MS/MS resolution was at 15,000, and the collision energy set at 10/30/60 in NCE mode. The spray voltage was set to 3.8 kV for positive mode and −3.4 kV for negative mode.

### 2.4 RNA extraction and library construction

Total RNA was extracted from tissues using TRIzol (Life Technologies) according to the manufacturer's instructions. RNA concentration and quality were measured with an Agilent 2100 Bioanalyzer. The samples with an RNA integrity number (RIN) > 5.0 were used for RNA-seq library preparation. The cDNA library was constructed using the TruSeq RNA Library Preparation Kit following the manufacturer's instructions (Illumina, San Diego, CA, USA) and checked with an Agilent 2100 Bioanalyzer. Subsequently, the Illumina HiSeq 2500 sequencing platform was used to sequence the library.

### 2.5 Real time quantitative PCR

To validate target gene expression in tissue samples, addition 4 HC, 6 LC, and 6 HCC samples were included for q-PCR. Briefly, total RNA was extracted using TRIzol (Life Technologies) according to the manufacturer's instructions. RNA concentration and quality were measured with Nanodrop one (Thermo Scientific). The reverse transcription reaction was performed with a cDNA synthesis kit (Transgen, AT341) for 15 min at 42°C. The q-PCR reaction was prepared following the manufacturer's instruction (Transgen, AQ601) and the value was calculated by the 2-ΔΔCT method. The primer sequences for AGXT2, CFHR4, KAT2A, MYL9 and GAPDH genes used in q-PCR are listed in Supplementary Table S1.

### 2.6 Data analysis

#### 2.6.1 Transcriptomic data analysis

Raw sequencing data in FASTQ format were firstly processed through in-house perl scripts. In this step, clean data (clean reads) were obtained by removing reads containing adapters, reads with poly-N sequences, and low-quality reads from the raw data. Simultaneously, Q20, Q30, GC content, and sequence duplication levels of the clean data were calculated. All downstream analyses were performed using high-quality clean data. The Homo sapiens GRCh38 reference genome (ftp://ftp.ensembl.org/pub/release87/fasta/homo_sapiens/dna/Homo_sapiens.GRCh38.dna.toplevel.fa.gz) was utilized as the reference for aligning the clean reads. Gene expression levels were quantified using fragments per kilobase of transcript per million fragments (FPKM) mapped. Differentially expressed genes (DEGs) were identified with *p*-value < 0.05 and |log2(fold change)|>1. Enrichment analysis of DEGs was performed using Gene Ontology (GO) and Kyoto Encyclopedia of Genes and Genomes (KEGG). The Gene Set Enrichment Analysis (GSEA) was employed for the enrichment analysis of all genes. These analyses were conducted utilizing the enrichplot package in R.

#### 2.6.2 Metabolomic data analysis

The raw data were converted to the mzXML format using ProteoWizard and processed with an in-house program, which was developed using R and was based on XCMS, for peak detection, extraction, alignment, and integration. Metabolite annotation was carried out using databases such as HMDB, MONA, METLIN, and an in-house MS2 database (BiotreeDB, Shanghai), with a cut-off score set at 0.3. Differentially abundant metabolites (DAMs) were selected based on a *p* < 0.05 (Student's *t*-test) and VIP >1 from orthogonal partial least squares discriminant analysis (OPLS-DA). Enrichment analysis of DAMs was performed using Kyoto Encyclopedia of Genes and Genomes (KEGG) by ggplot2 package in R.

For mass spectrometry imaging (MSI), the raw data files were converted into.cdf format and analyzed using homemade imaging software (MassImager, Beijing, China), following a previously reported method (He et al., 2018). Metabolites were annotated using the pySM pipeline and an in-house SmetDB database (Lumingbio, Shanghai). OPLS-DA and partial least-squares discriminant analysis were used to identification and selection of DAMs, based on *p* < 0.05 (Student's *t-*test and one-way ANOVA Dunnett's test) and VIP > 1. Enrichment analysis of DAMs was performed using Kyoto Encyclopedia of Genes and Genomes (KEGG) by ggplot2 package in R.

#### 2.6.3 Statistical analysis

All statistical analyses were conducted using GraphPad Prism 9.0 (San Diego, CA, USA) and R software (v4.1.0). Quantitative results are expressed as the mean ± standard deviation. Student's *t*-test was utilized for comparisons between two groups, while one-way ANOVA was employed for multiple group comparisons. Statistical significance is indicated by *p* < 0.05.

## 3 Results

### 3.1 Characteristics of recruited participants

This study included 12 liver cirrhosis (LC), eight hepatocellular carcinoma (HCC), and seven healthy control liver tissue samples for transcriptomic and untargeted metabolomic studies. Clinical data for these participants are summarized in Figures 1D–F. All participants tested positive for HBsAg or HBV copy number, indicating that HBV infection was a key risk factor for liver cirrhosis and cancer. Elevated levels of alanine transaminase (ALT), aspartate aminotransferase (AST) and total bile acid (TB) level were observed in most patients. Notably, the carcinoembryonic antigen (CEA) and α-fetoprotein (AFP) levels increased in the HCC group. Conversely, total protein (TP) and cholinesterase (CHE) levels significantly decreased in most patients. The Child-Pugh and MELD scores calculated based on clinical data and ultrasound results, demonstrated the liver condition of the participants and confirmed cirrhosis and tumor formation.

### 3.2 Alternation of gene expression in liver cirrhosis

**Figure 2:**
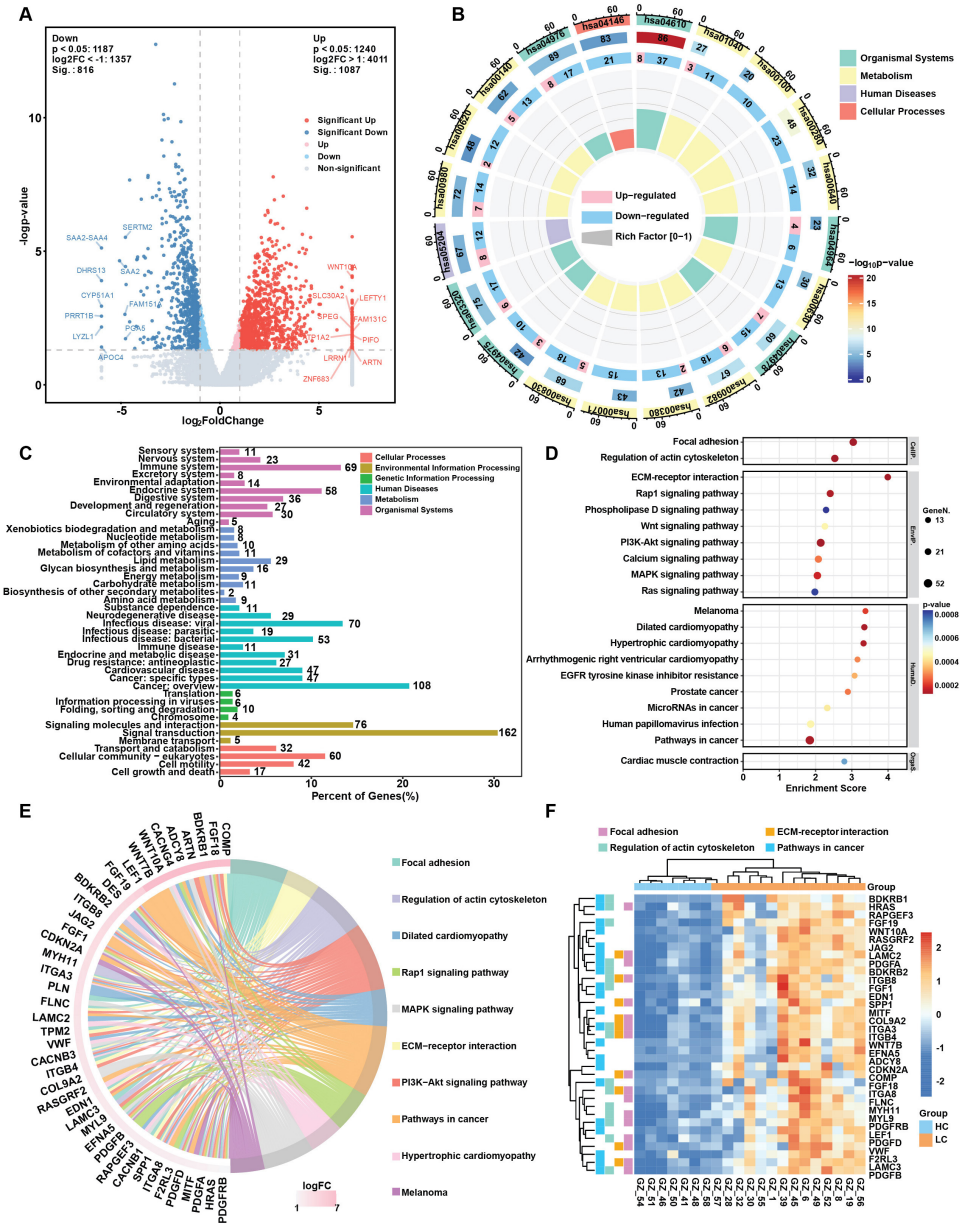
Changes in gene expression in the HBV related liver cirrhosis. **(A)** Volcano plot of differentially expressed genes (DEGs) in liver cirrhosis. **(B)** Circos map of DEGs. The circles from inside to outside represent the enrichment factor, the ratio of up- and downregulated genes, background genes, *p*-value and KEGG level 1 classification. **(C)** KEGG pathway classification of upregulated genes in liver cirrhosis. **(D)** Top 20 KEGG enrichment items for upregulated genes in liver cirrhosis. **(E)** Circos plot of the top upregulated genes and related KEGG pathways in liver cirrhosis. **(F)** Heatmap of selected upregulated DEGs and key pathways involved in HBV related liver cirrhosis.

Liver tissue samples from the HC, LC and HCC groups were subjected to transcriptomic studies, as shown in the workflow in Figure 1A. Principal component analysis (PCA) results revealed three distinct clusters for each group, with the HC group clearly separated from the LC and HCC groups (Figure 1B), validating the accuracy of sequencing and the differing conditions among the groups. Most of the detected genes were shared among the three groups (17,485), with only a few hundred genes unique to each group (Figure 1C). Based on the |Log2FC| > 1 and *p* < 0.05, DEGs were identified in LC and HCC groups compared to the HC group. In the LC group, 1,087 genes were significantly upregulated and 816 were downregulated (Figure 2A). The KEGG enrichment analysis of all DEGs indicated that the pathways were mainly enriched in metabolism, including the biosynthesis of unsaturated fatty acids, fatty acid degradation and steroid biosynthesis (Figure 2B). Both upregulated and downregulated genes were primarily enriched in KEGG classifications related to lipid metabolism cancer overview and viral infection (Figure 2C; Supplementary Figure S1A). The top enriched pathways for upregulated genes included focal adhesion, regulation of actin cytoskeleton, ECM-receptor interaction and pathway in cancer, indicating changes in liver structure from the normal hepatic lobule to the pseudo lobule (Figures 2D, E). Highly expressed genes in the LC group included COL9A2, LAMC2, LAMC3, and MYL9, which are related to ECM formation (Figure 2F). In contrast downregulated genes were enriched in complement and coagulation cascades, biosynthesis of unsaturated fatty acids and fatty acid degradation pathways (Supplementary Figure S1B). The key downregulated genes included the CFHR family genes (CFHR3-5), which are involved in complement activation, and ACSL1, ACSL3, and ACSL4, which regulate fatty acid synthesis and also metabolite profile-associated enzyme AGXT2, ACSS2, and ACSS3. The gene set enrichment analysis (GSEA) also highlighted focal adhesion and Hedgehog signaling pathways, which are associated with tumorigenesis. Overall, gene alternations in the LC group facilitated disease progression and the formation of pseudo lobule.

### 3.3 Gene expression alternation participate tumorigenesis

**Figure 3:**
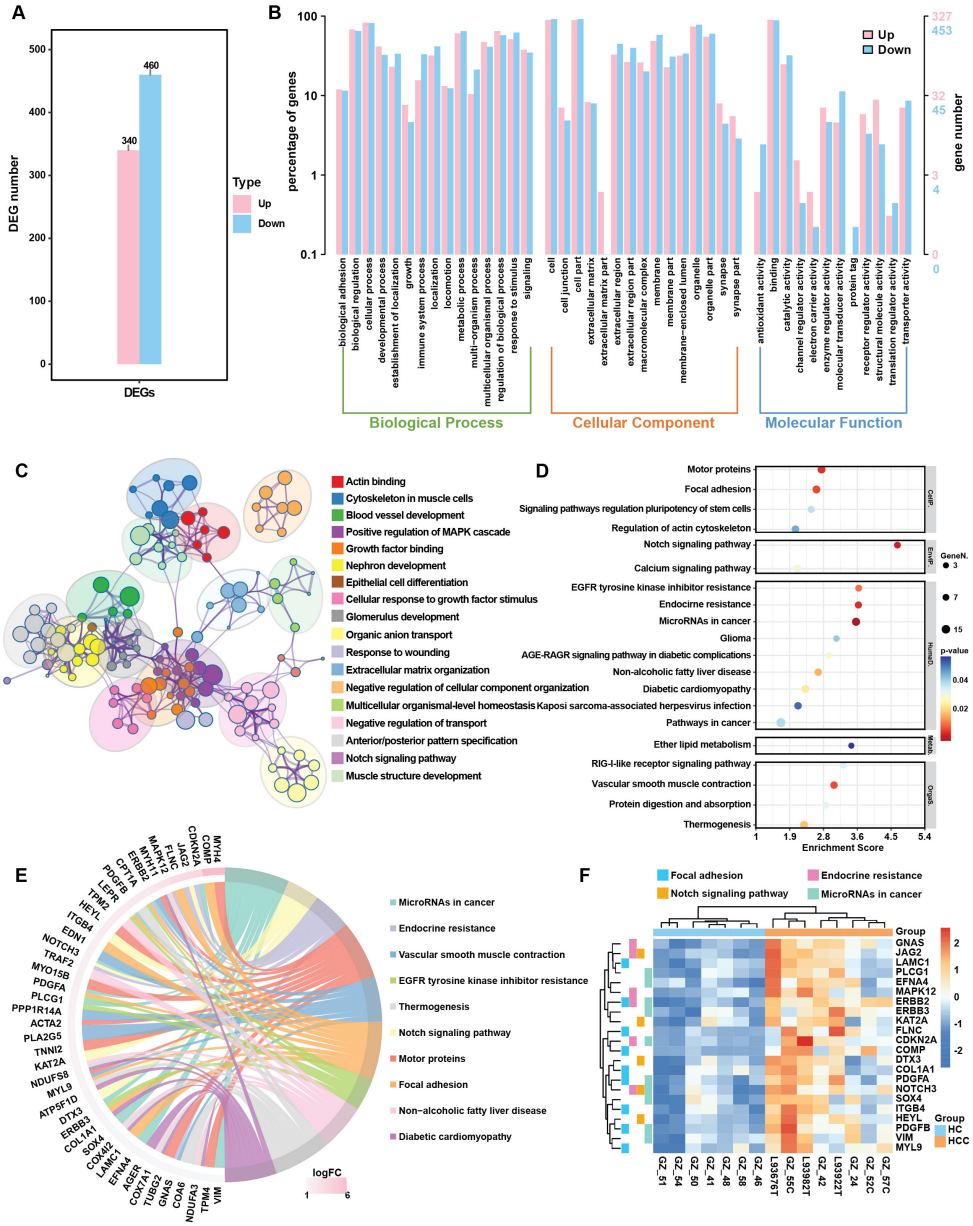
DEG analysis in the HBV related HCC group. **(A)** Bar plot of DEGs in HCC group. **(B)** GO enrichment analysis of up- and downregulated genes in HCC group. **(C)** Metascape analysis compared the signal pathways of DEGs in HCC group. **(D)** Top 20 KEGG enrichment items for upregulated genes in the HCC group. **(E)** Circos plot of the top upregulated genes and related KEGG pathways in the HCC group. **(F)** Heatmap of selected upregulated DEGs and key regulated pathways in the HCC.

To assess whether gene expression changes in HCC followed a similar trend, we compared DEGs and enrichment pathways in the HCC group. In HCC, 340 genes were upregulated and 460 were downregulated (Figure 3A). KEGG enrichment analysis of all DEGs revealed that the pathways related to metabolism, including biosynthesis of unsaturated fatty acids, fatty acid degradation and steroid biosynthesis, were similarly enriched in HCC group (Figure 3B). The top KEGG classifications for both upregulated and downregulated genes were lipid metabolism, cancer overview and viral infection, similar to those enriched in the LC group (Figure 3C; Supplementary Figure S2A). These findings suggest that continued HBV infection plays a key role in tumorigenesis. The top enriched pathways for the upregulated genes included focal adhesion, regulation of actin cytoskeleton and pathway in cancer (Figure 3D), as well as the key upregulated genes included LAMC1, MYL9, COL1A, and KAT2A which, regulate ECM formation and actin skeleton (Figures 3E, F). Downregulated genes included CFHR family genes (CFHR3-4) and complement family genes (C4A-B, C6, C9), which are involved in complement and coagulation cascades (Supplementary Figures S2C, D). The q-PCR validation indicated the significant upregulation of MYL9 and KAT2A expression and downregulation of CHFR4 and AGXT2 (Supplementary Figure S3).

### 3.4 Disease progression and gene expression patterns

**Figure 4:**
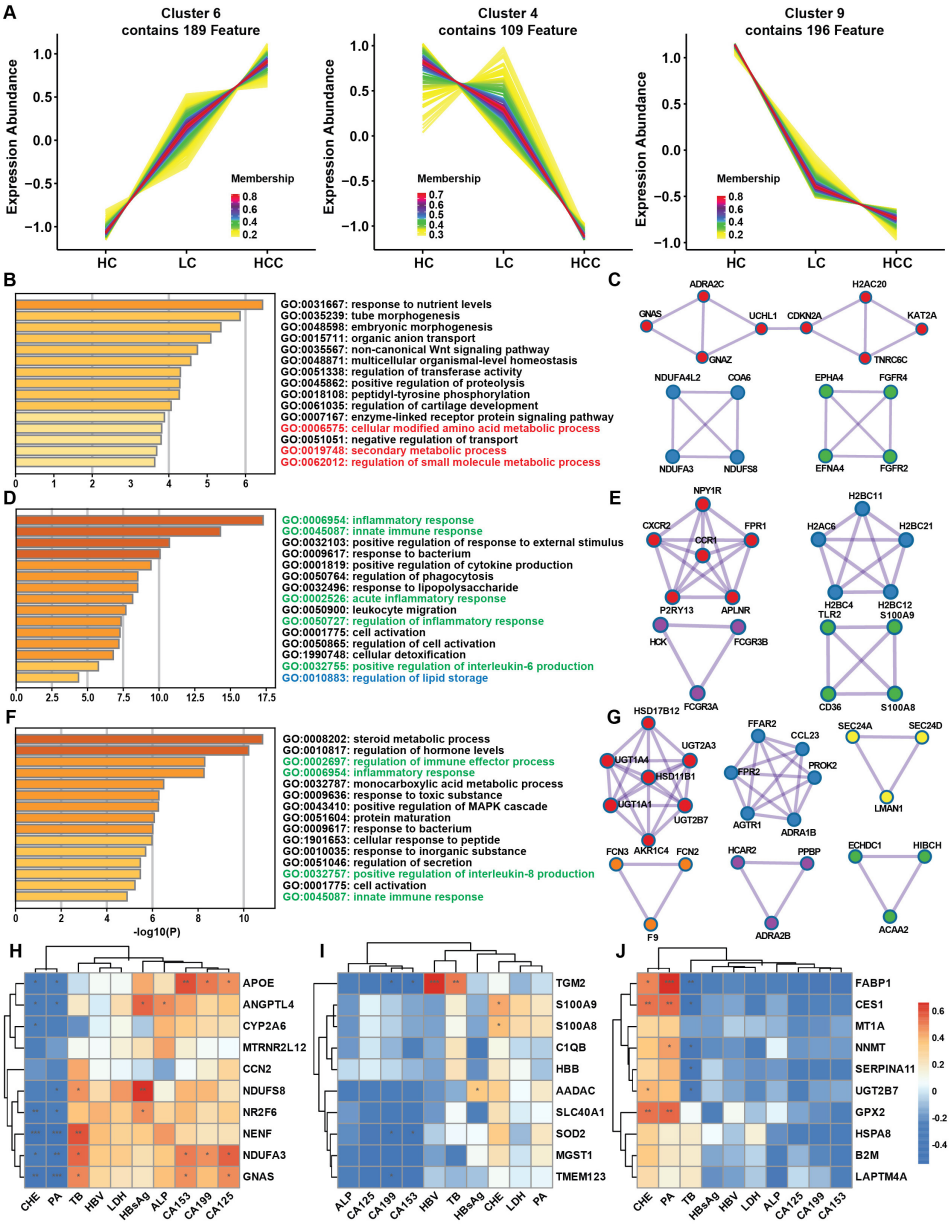
Gene expression pattern during disease progression and clinical correlation. **(A)** Time series analysis of gene expression patterns among the HC, LC and HCC groups. **(B)** Metascape enrichment analysis of genes with increasing trends. **(C)** Protein-protein interaction (PPI) analysis highlighting hub genes among the genes with increasing trends. **(D, F)** Metascape enrichment analysis of genes with decreasing trends. **(E, G)** PPI analysis of the hub genes among the genes with decreasing trends. **(H–J)** Correlations of increasing **(H)** and decreasing **(I, J)** trend genes with clinical data. *P < 0.05, **P < 0.01, ***P < 0.001.

After separately comparing gene expression alternations in the LC and HCC groups, we conducted the time series analysis to identify genes with continued rising or decreasing expression trend to elucidate the effect of disease progression on gene expression patterns. Time series analysis identified nine clusters of genes, with cluster 6 (189 genes) showing a continued increase in expression, and cluster 4 (109 genes) and cluster 9 (196 genes) exhibiting a decreasing trend (Figure 4A). GO enrichment analysis of the genes with increased expression indicated enrichment in cellular component (CC) category with extracellular space, collagen trimer, collagen-containing extracellular matrix pathways (Supplementary Figure S4A). Combining the two continuously decreasing clusters, the GO enrichment results revealed pathways such as immune response and inflammatory response in the biological process (BP) category (Supplementary Figure S4B). Metascape analysis was used to identify protein-protein interaction (PPI) and hub genes among genes with both increasing and decreasing expression patterns. In the increasing expression pattern, the enriched pathways included amino acid metabolism and secondary metabolic process (Figure 4B). Three MCODEs are involved in the regulation of kinase activity, regulation of transferase activity and peptidyl-tyrosine phosphorylation. Hub gene such as KAT2A was also among the top increased genes in both LC and HCC groups (Figure 4C). In clusters 4 and 9, genes showed a decreasing expression trend, with GO enrichment analysis indicating similar results concentrating on inflammatory response, immune response and lipid metabolism (Figures 4D, F). The MCODEs in cluster 4 were related to immune and inflammatory response, while cluster 9 was associated with steroid metabolism process and glucuronate metabolic process (Figures 4E, G). The expression of several hub genes, such as CXCR2 and TLR2 was correlated with immune response. To test whether the gene expression pattern correlated with clinical data and had the potential diagnostic value, we analyzed the correlation between genes with increasing or decreasing trends and clinical data. The top 10 correlated genes from increasing expression pattern were mostly negatively correlated with prealbumin (PA) and cholinesterase (CHE), and positively correlated with total bile acid (TB). Additionally, APOE, NDUFA3 and GNAS were positively correlated with CA153, CA199 and CA125 (Figure 4H). Conversely, cluster 4 genes had less correlation with the clinical data, while genes from cluster 9 had opposite correlations with PA, CHE and TB (Figures 4I, J). These results demonstrated that disease progression affects gene expression patterns, leading to continuous alternation of key genes that regulate immune response and metabolic processes.

### 3.5 Metabolic alternations in LC and HCC

**Figure 5:**
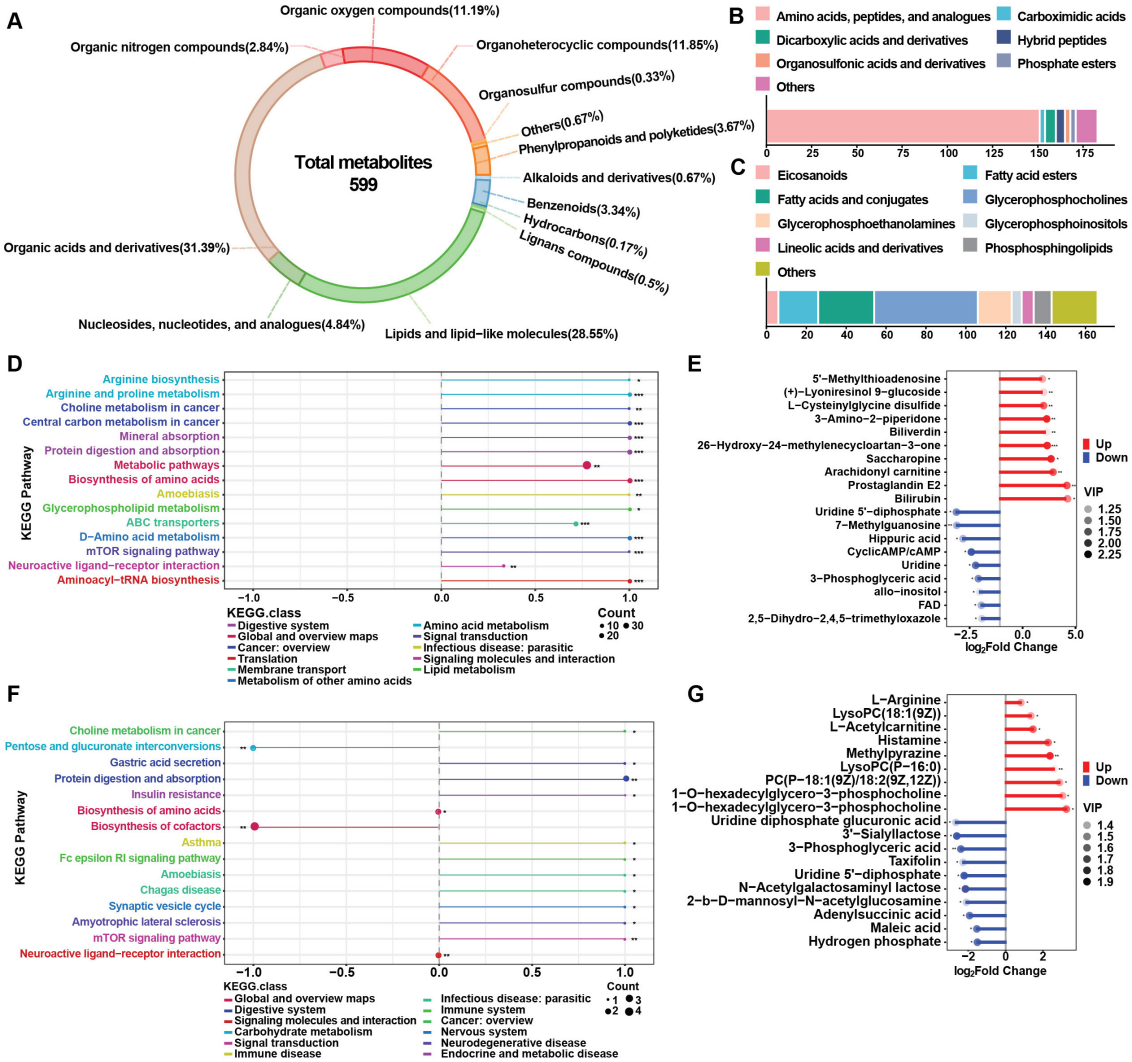
Metabolite distribution in the HBV related LC and HCC groups. **(A)** Total metabolites and their classification. **(B)** Subclassification of organic acids and derivatives. **(C)** Subclassification of lipids and lipid-like molecules. **(D, F)** Differential abundance scores of metabolites in the LC **(D)** and HCC **(F)** groups. **(E, G)** Analysis of the top regulated metabolites in the LC **(E)** and HCC **(G)** groups. *P < 0.05, **P < 0.01, ***P < 0.001.

**Figure 6:**
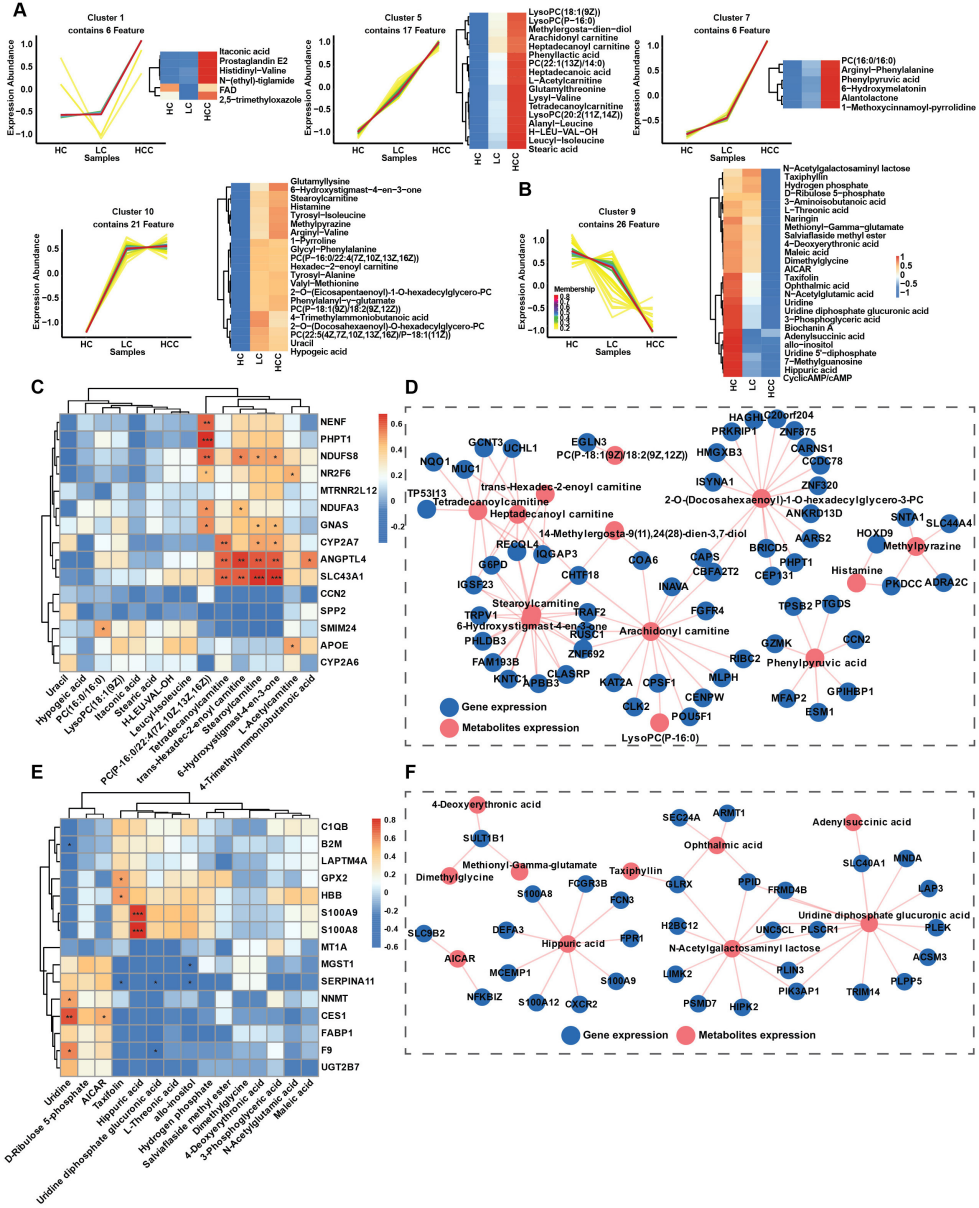
Metabolite expression patterns during disease progression and gene correlation. **(A, B)** Time series analysis of metabolite expression patterns among the HC, LC and HCC groups, metabolites with increasing trends in expression metabolites **(A)**, and metabolites with decreasing trends in expression **(B)**. **(C, E)** Correlations between metabolites and gene expression for increasing **(C)** and decreasing trends **(E)**. **(D, F)** Interaction network between metabolites and genes expression (*R* > 0.75), for increasing **(D)**, and decreasing trends **(F)**. *P < 0.05, **P < 0.01, ***P < 0.001.

The transcriptomic study indicated the alternations of several metabolic pathways, including the biosynthesis of unsaturated fatty acids, fatty acid degradation and amino acid metabolism. Therefore, an untargeted metabolomic study was conducted to determine changes in metabolic pathways and key metabolite alternation patterns during disease progression and to elucidate the correlation between metabolites and gene expression. In total 599 metabolites were identified using LC-MS/MS and were divided into 13 classes. The top two classes were organic acids and derivatives (31.39%) and lipids and lipids-like molecules (28.55%) (Figure 5A). These two classes were further divided into 7 and 9 subclasses, respectively, with amino acid, peptides and analogs and glycerophosphocholines being the most abundant (Figures 5B, C). Based on VIP > 1 and *p* < 0.05, differentially abundant metabolites (DAMs) were selected for the LC and HCC groups compared to the HC group. Differential abundance scores (DA scores) were calculated for DAMs in the LC and HCC groups. The results indicated significant upregulation in the choline metabolism in cancer pathway for both the LC and HCC groups, as well as glycerophospholipids metabolism in the LC group (Figures 5D, F). The top 10 up- and downregulated metabolites were amino acids and glycerophospholipids, such as PC and LysoPC (Figures 5E, G). To evaluate whether the metabolites had similar expression pattern to those of genes, we conducted the time series analysis of the metabolites. The results revealed that four clusters (clusters 1, 5, 7, and 10) exhibited an increasing expression pattern, while one cluster (cluster 9) exhibited a decreasing pattern. The metabolites with increased expression pattern were PCs, lysoPCs and carnitines, whereas those with decreased expression were mostly organic acids (Figures 6A, B).

### 3.6 Correlation between gene expression and metabolism

**Figure 7:**
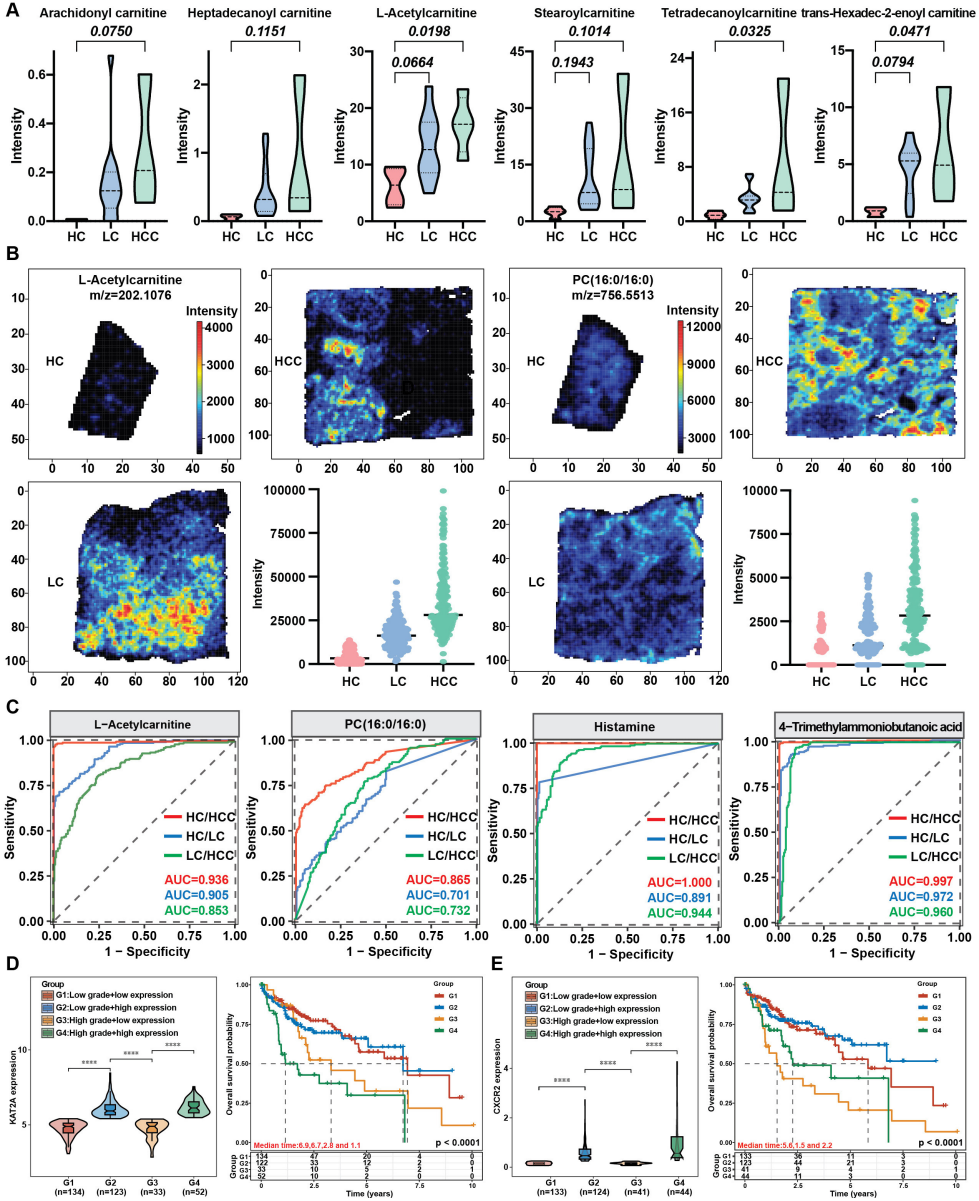
Metabolite spatial distribution changes during disease progression and the prediction value of selected metabolites and genes. **(A)** Expression of carnitine metabolites with disease progression. **(B)** Spatial distribution of metabolites with increasing trends. **(C)** AUC curve of key metabolites. **(D, E)** Expression and survival curves of increasing **(D)** and decreasing **(E)** trend genes from the TCGA database. ****P < 0.0001.

To investigate whether the gene expression patterns affected metabolite expression, we analyzed correlations between up-and downregulated genes and metabolites with similar expression patterns, clustering correlation heatmaps and correlation network maps were generated. The clustering correlation heatmap with signs and correlation network were created using the Cloud.oebiotech tools at https://cloud.oebiotech.com/#/bio/tools. The clustering correlation heatmap was selected based on Pearson's *r* > 0.6. For the upregulated pattern, genes such as NR2F6, APOE, NDUFS8, CYP2A7, ANGPTL4, and SLC43A1 were significantly positively correlated with several carnitine metabolites, including L-acetylcarnitine, stearoylcarnitine, trans-hexadec-2-enoylcarnitine and tetradecanoylcarnitine (Figure 6C). Conversely, in the downregulated pattern, S100A8 and S100A9 were closely correlated with hippuric acid, and NNMT, CES1, and F9 were positively correlated with uridine (Figure 6E). A correlation network between gene and metabolite expression was established based on Pearson's *r* > 0.75 and *p* < 0.05. For the upregulated pattern, the metabolites were mainly carnitine, PC, lysoPC and histamine (Figure 6D). Organic acids predominated in the downregulated network (Figure 6F). Given the significant correlation of several carnitine metabolites with the upregulated expression pattern, all identified carnitine metabolites were evaluated. Among the six identified carnitine metabolites, L-acetylcarnitine, trans-hexadec-2-enoylcarnitine and tetradecanoylcarnitine exhibited significantly increased expression with disease progression (Figure 7A). Meanwhile, genes regulating carnitine metabolism, such as CPT1A and CPT1C, exhibited a similar increasing expression pattern (Supplementary Figure S5A). To further validate expression patterns in relation to disease progression, mass spectrometry imaging (MSI) was employed to assess the spatial distribution of upregulated metabolites. In combination with hematoxylin-eosin staining, regions encompassing hepatic lobules, pseudo lobules, and tumor areas were selected across HC, LC, and HCC groups. The four detected metabolites L-acetylcarnitine, PC (16:0/16:0), histamine, and 4-trimethylammoniobutanoic acid exhibited a progressive increase in concentration from hepatic lobules to tumor regions (Figure 7B; Supplementary Figure S5B). These findings indicate that, with disease progression, lesion areas demonstrate significant alterations in the spatial distribution of these metabolites. The predictive value of these four metabolites was evaluated using receiver operating characteristic (ROC) curves to distinguish different disease stages. With the exception of PC (16:0/16:0), three metabolites achieved AUC > 0.85 for distinguishing the HC, LC and HCC groups (Figure 7C). Additionally, KAT2A and CXCR2 selected from up- and downregulated expression patterns and closely correlated with metabolites, were evaluated for prognosis in HCC. TCGA database analysis revealed that KAT2A expression was significantly increased, while CXCR2 had the opposite expression pattern (Supplementary Figure S5C). High KAT2A expression in high-grade patients significantly reduced survival time from a median of 6.9 years (low grade and low expression) to 1.1 years (Figure 7D). Conversely, high-grade patients with lower CXCR2 expression exhibited shorter survival times, ranging 5.6–1.5 years (Figure 7E). Therefore, metabolites and genes with up- or downregulated expression patterns have the potential to predict clinical outcomes and can be further developed as diagnostic markers for the early detection of HBV related LC and HCC.

## 4 Discussion

In this study, we employed a multiomics approach to comprehensively analyze the molecular landscape of liver cirrhosis (LC) and hepatocellular carcinoma (HCC). Our transcriptomic analysis revealed substantial differences in gene expression patterns across the healthy control (HC) and disease groups. Most genes were commonly expressed across all groups (17,485), and each group exhibited unique gene expression profiles, indicating dynamic alterations during disease progression. Differential expression analysis identified 1,903 and 800 genes as significantly altered in LC and HCC, respectively, with distinct upregulation (e.g., 1,087 in LC and 340 in HCC) and downregulation patterns (e.g., 816 in LC and 460 in HCC). These findings align with previous studies highlighting the extensive transcriptional changes associated with chronic hepatitis B infection (Yang et al., 2022). Moreover, the DEGs from tissues general have larger number compared to DEGs from plasmas (Kuang et al., 2024). Enrichment analysis of KEGG class revealed that genes related to the immune system, lipid metabolism, pathway in cancer and viral infection were particularly affected, emphasizing their roles in tumor development and the viral response. A study comparing chronic hepatitis B (CHB) and acute-on-chronic liver failure (ACLF) found that immunometabolism were significantly enriched in downregulation pathways during the progression from CHB to ACLF, including amino acid metabolism, fatty acid metabolism, and peroxisome metabolism (Yang et al., 2022). Our results revealed that the biosynthesis of unsaturated fatty acids, fatty acids degradation and peroxisome pathways were also enriched among the downregulated genes. In addition, many studies have confirmed that liver cirrhosis is associated with decreased both circulating lipid and hepatic lipid accumulation (Chrostek et al., 2014; Meikle et al., 2015), as supported by the downregulation of fatty acid biosynthesis pathways. It has been reported that the liver produces most complement factors, such as C3 and C4, which are produced by hepatocytes (Thorgersen et al., 2019). Chronic hepatitis B infection disrupts both the innate and adaptive immune systems (Albillos et al., 2022), which contributes to liver cirrhosis due to decreased production of complement proteins like C3 and C4. Notably, the complement and coagulation cascade, critical for liver homeostasis, showed downregulation in both LC and HCC, indicating impaired liver function and disease progression. A broad range of CHB patients with different disease severities showed a decreasing trend in C3 level with disease progression, suggesting that the liver injury affects on complement system (Chen et al., 2023). The production of complement proteins, such as albumin, can be reduced in patients with HBV related liver cirrhosis (Homann et al., 1997). Our clinical data corroborate these findings, showing reductions in total protein, albumin, and globulin levels. In the contrast, enrichment analysis revealed that upregulated genes in both LC and HCC groups were enriched in pathways related to cell adhesion, actin cytoskeleton regulation, and extracellular matrix (ECM) interactions, which are crucial for ECM remodeling. The progression of cirrhosis changes the structure of the liver, replacing normal cells with scar tissue (Pellicoro et al., 2014; Albillos et al., 2022). Hepatic stellate cells (HSCs) play a crucial role in the development of liver fibrosis and cirrhosis. When the liver is injured, these cells become active and transform into myofibroblast-like cells, which produce large amounts of extracellular matrix (ECM), including proteins like collagen and laminins (Hintermann and Christen, 2019). The deposition of ECM proteins, leads to scar tissue formation and distorts the liver's structure, resulting in the characteristic pseudolobules observed in cirrhosis (Cui et al., 2014). Overall, changes in various pathways, such as decreased fatty acid metabolism and complement production, along with increased ECM remodeling, drive the structural changes in the liver, promoting cirrhosis and tumor formation.

Further analysis focused on identifying key genes and pathways associated with the transition from LC to HCC. Upregulated genes in both conditions included ECM-related genes (COL1A1, COL9A2, LAMC1, LAMC2, LAMC3), reinforcing their role in ECM remodeling and disease progression. The downregulated genes included those in the CFHR, complement, and acyl-CoA synthetase long-chain families, indicating suppressed complement function and fatty acid synthesis. Through time series analysis, we identified clusters of genes with increasing or decreasing expression patterns. Hub genes like KAT2A was prominent in the upregulated clusters and were closely related to critical metabolic processes. Several studies have indicated that KAT2A is overexpression in multiple cancers compared with adjacent tissues, including liver cancer (Majaz et al., 2016), colon adenocarcinoma tissues (Yin et al., 2015), and non-small cell lung cancer tissues (Chen et al., 2013). Conversely, decreasing KAT2A expression can significantly reduce the proliferation and migration of cancer cells and the growth of xenograft tumors (Zhao et al., 2018; Lin et al., 2022). In the present study, KAT2A expression in the TCGA database indicated that KAT2A overexpression in HCC was linked to poor survival time. A study showed that KAT2A promotes HBV transcription through covalently closed circular DNA (cccDNA)-bound succinylated histone H3K79 (Qin et al., 2022). Taken together, these studies suggest KAT2A can be potential as a prognostic biomarker for the disease progression and needs further validation.

Metabolomic profiling identified key metabolites and pathways altered in liver cirrhosis and hepatocellular carcinoma, provided insights into the metabolic reprogramming associated with diseases and complemented transcriptomic findings by identifying significant alteration of metabolites. Our metabolomic profiling revealed significant changes in metabolites associated with organic acids and derivatives (31.39%) and lipids and lipids-like molecules (28.55%). Key metabolic pathways, such as choline metabolism and glycerophospholipid metabolism, were prominently dysregulated in both LC and HCC, mirroring the transcriptomic results and underscoring their importance in disease pathogenesis. The expression patterns of metabolites were evaluated and the networks between the same expression patterns of metabolites and genes were established. We identified key metabolites like carnitines, closely correlated with genes such as KAT2A, CYP2A7, ANGPTL4, and SLC43A1. L-carnitine is essential for fatty acid transport into mitochondria for oxidation. Many studies have demonstrated stepwise changes in either serum or plasma L-carnitine levels in patients with HBV related cirrhosis and HCC (Gong et al., 2017; Gu et al., 2021). Meanwhile, L-carnitine levels were positively correlated with ALT, AST, and bilirubin levels (Gu et al., 2021), indicating their potential as diagnostic markers for HBV-related diseases.

At present, early diagnosis of HCC remains a difficult problem. The commonly used tumor marker, AFP, has limited sensitivity and specificity, with most patients being diagnosed at a late stage (Toyoda et al., 2015). We identified four metabolites that increased from LC to HCC and showed significant spatial distribution within tissues as the potential biomarkers for diagnosis. Three metabolites (L-acetylcarnitine, histamine, and 4-trimethylammoniobutanoic acid) show strong discrimination between LC, HCC, and healthy controls (AUC > 0.9) and between LC and HCC (AUC > 0.85). Previous research has indicated that the levels of certain unsaturated long-chain acylcarnitines (AC14:1 and AC18:1), such as acetylcarnitine, rise as liver fibrosis and HCC progress and acylcarnitine 10:2 isomer2 and acylcarnitine 12:2 isomer2 exhibit high diagnostic capability with AUC > 0.95 (Abbass et al., 2024; Wu L. J. Y. et al., 2024). In addition, high levels of histamine were linked to circulatory dysfunction in advanced chronic liver disease patients and independently associated with increased risks of acute-on-chronic liver failure or liver-related death (Schwarz et al., 2024). These findings suggest that multiple metabolites could effectively differentiate between HBV related LC and HCC and further investigations are needed to determine their diagnostic value.

## Data Availability

The datasets generated and analyzed during the current study are available in the NCBI repository. The data can be accessed using the following accession numbers: PRJNA1194226 and PRJNA1194218.

## References

[B1] Abbass A. Sheashaey A. E. El Fert A. Obada M. Abdelsameea E. Abdel-Samiee M.. (2024). Exploring the prognostic significance of blood carnitine and acylcarnitines in hepatitis C virus-induced hepatocellular carcinoma. Egypt. Liver J. 14:19. 10.1186/s43066-024-00322-x

[B2] Albillos A. Martin-Mateos R. Van der Merwe S. Wiest R. Jalan R. Alvarez-Mon M. (2022). Cirrhosis-associated immune dysfunction. Nat. Rev. Gastroenterol. Hepatol. 19, 112–134. 10.1038/s41575-021-00520-7 34703031

[B3] Cai F. F. Song Y. N. Lu Y. Y. Zhang Y. Y. Hu Y. Y. Su S. B. (2020). Analysis of plasma metabolic profile, characteristics and enzymes in the progression from chronic hepatitis B to hepatocellular carcinoma. Aging 12, 14949–14965. 10.18632/aging.103554 32701483 PMC7425494

[B4] Chen C. Yuan Z. Li W. X. Fei L. Ji L. J. Huang Q.. (2023). Complement C3 facilitates stratification of stages of chronic hepatitis b and signifies development of acute-on-chronic liver failure in acute decompensated cirrhosis. Adv. Ther. 40, 1171–1186. 10.1007/s12325-022-02416-7 36652176 PMC9848025

[B5] Chen L. Wei T. Y. Si X. X. Wang Q. Q. Li Y. Leng Y.. (2013). Lysine acetyltransferase GCN5 potentiates the growth of non-small cell lung cancer via promotion of E2F1, cyclin D1, and cyclin E1 expression. J. Biol. Chem. 288, 14510–14521. 10.1074/jbc.M113.458737 23543735 PMC3656305

[B6] Cheng Y. He J. Zuo B. He Y. (2024). Role of lipid metabolism in hepatocellular carcinoma. Discov. Oncol. 15:206. 10.1007/s12672-024-01069-y 38833109 PMC11150225

[B7] Chrostek L. Supronowicz L. Panasiuk A. Cylwik B. Gruszewska E. Flisiak R. (2014). The effect of the severity of liver cirrhosis on the level of lipids and lipoproteins. Clin. Exp. Med. 14, 417–421. 10.1007/s10238-013-0262-5 24122348 PMC4213382

[B8] Cui X. D. Zhang X. Y. Yin Q. L. Meng A. X. Su S. J. Jing X.. (2014). F-actin cytoskeleton reorganization is associated with hepatic stellate cell activation. Mol. Med. Rep. 9, 1641–1647. 10.3892/mmr.2014.2036 24626324 PMC4020483

[B9] Fan C. F. Kam S. Ramadori P. (2021). Metabolism-associated epigenetic and immunoepigenetic reprogramming in liver cancer. Cancers 13:5250. 10.3390/cancers13205250 34680398 PMC8534280

[B10] Gajos-Michniewicz A. Czyz M. (2024). WNT/beta-catenin signaling in hepatocellular carcinoma: the aberrant activation, pathogenic roles, and therapeutic opportunities. Genes Dis. 11, 727–746. 10.1016/j.gendis.2023.02.050 37692481 PMC10491942

[B11] Gong Z. G. Zhao W. J. Zhang J. B. Wu X. Hu J. Yin G. C.. (2017). Metabolomics and eicosanoid analysis identified serum biomarkers for distinguishing hepatocellular carcinoma from hepatitis B virus-related cirrhosis. Oncotarget 8, 63890–63900. 10.18632/oncotarget.19173 28969038 PMC5609970

[B12] Gu S. Q. Fu X. Ye G. F. Chen C. C. Li X. Y. Zhong S. H.. (2021). High L-carnitine levels impede viral control in chronic hepatitis B virus infection. Front. Immunol. 12:649197. 10.3389/fimmu.2021.649197 34234772 PMC8255973

[B13] He J. M. Huang L. J. Tian R. T. Li T. G. Sun C. L. Song X. W.. (2018). MassImager: a software for interactive and in-depth analysis of mass spectrometry imaging data. Anal. Chim. Acta 1015, 50–57. 10.1016/j.aca.2018.02.030 29530251

[B14] He M. J. Pu W. Wang X. Zhong X. Zhao D. Zeng Z.. (2022). Spatial metabolomics on liver cirrhosis to hepatocellular carcinoma progression. Cancer Cell Int. 22:366. 10.1186/s12935-022-02775-9 36419080 PMC9686114

[B15] Hintermann E. Christen U. (2019). The many roles of cell adhesion molecules in hepatic fibrosis. Cells 8:1503. 10.3390/cells8121503 31771248 PMC6952767

[B16] Homann C. Varming K. Hogasen K. Mollnes T. E. Graudal N. Thomsen A. C.. (1997). Acquired C3 deficiency in patients with alcoholic cirrhosis predisposes to infection and increased mortality. Gut 40, 544–549. 10.1136/gut.40.4.544 9176087 PMC1027133

[B17] Hoxhaj G. Manning B. D. (2020). The PI3K-AKT network at the interface of oncogenic signalling and cancer metabolism. Nat. Rev. Cancer 20, 74–88. 10.1038/s41568-019-0216-7 31686003 PMC7314312

[B18] Hsu Y. C. Huang D. Q. Nguyen M. H. (2023). Global burden of hepatitis B virus: current status, missed opportunities and a call for action. Nat. Rev. Gastroenterol. Hepatol. 20, 524–537. 10.1038/s41575-023-00760-9 37024566

[B19] Kuang X. Li J. Xu Y. Yang L. Liu X. Yang J.. (2024). Transcriptomic and metabolomic analysis of liver cirrhosis. Comb. Chem. High Throughput Screen. 27, 922–932. 10.2174/1386207326666230717094936 37461343 PMC11092553

[B20] Liang H. Song K. (2023). Comprehensive metabolomics and transcriptomics analysis reveals protein and amino acid metabolic characteristics in liver tissue under chronic hypoxia. PLoS ONE 18:e0291798. 10.1371/journal.pone.0291798 37747892 PMC10519603

[B21] Lin S. Qiu L. Liang K. Zhang H. Xian M. Chen Z.. (2022). *KAT2A/E2F1* promotes cell proliferation and migration via upregulationg the exression of *UBE2C* in pan-cancer. Genes 13:1817. 10.3390/genes13101817 36292703 PMC9602169

[B22] Llovet J. M. Kelley R. K. Villanueva A. Singal A. G. Pikarsky E. Roayaie S.. (2021). Hepatocellular carcinoma. Nat. Rev. Dis. Prim. 7:6. 10.1038/s41572-020-00240-3 33479224 PMC13537433

[B23] Majaz S. Tong Z. W. Peng K. S. Wang W. Ren W. J. Li M.. (2016). Histone acetyl transferase GCN5 promotes human hepatocellular carcinoma progression by enhancing AIB1 expression. Cell Biosci. 6:47. 10.1186/s13578-016-0114-6 27486509 PMC4969657

[B24] Meikle P. J. Mundra P. A. Wong G. Rahman K. Huynh K. Barlow C. K.. (2015). Circulating lipids are associated with alcoholic liver cirrhosis and represent potential biomarkers for risk assessment. PLoS ONE 10:e0130346. 10.1371/journal.pone.0130346 26107182 PMC4479371

[B25] Nartey Y. A. Antwi S. O. Bockarie A. S. Hiebert L. Njuguna H. Ward J. W.. (2022). Mortality burden due to liver cirrhosis and hepatocellular carcinoma in Ghana; prevalence of risk factors and predictors of poor in-hospital survival. PLoS ONE 17:e0274544. 10.1371/journal.pone.0274544 36099308 PMC9469955

[B26] Pellicoro A. Ramachandran P. Iredale J. P. Fallowfield J. A. (2014). Liver fibrosis and repair: immune regulation of wound healing in a solid organ. Nat. Rev. Immunol. 14, 181–194. 10.1038/nri3623 24566915

[B27] Pinzani M. Rosselli M. Zuckermann M. (2011). Liver cirrhosis. Best Pract. Res. Clin. Gastroenterol. 25, 281–290. 10.1016/j.bpg.2011.02.009 21497745

[B28] Pu W. Wang X. Zhong X. Zhao D. Zeng Z. Cai W.. (2023). Dysregulation of lipid metabolism in the pseudolobule promotes region-specific autophagy in hepatitis B liver cirrhosis. Hepatol. Commun. 7:187. 10.1097/HC9.0000000000000187 37486962 PMC10368385

[B29] Qin Y. P. Yu H. B. Yuan S. Y. Yang Z. Ren F. Wang Q.. (2022). KAT2A promotes hepatitis B virus transcription and replication through epigenetic regulation of cccDNA minichromosome. Front. Microbiol. 12:795388. 10.3389/fmicb.2021.795388 35140694 PMC8818952

[B30] Schwarz M. Simbrunner B. Jachs M. Hartl L. Balcar L. Bauer D.. (2024). High histamine levels are associated with acute-on-chronic liver failure and liver-related death in patients with advanced chronic liver disease. Liver Int. 44, 2904–2914. 10.1111/liv.16056 39136222

[B31] Thorgersen E. B. Barratt-Due A. Haugaa H. Harboe M. Pischke S. E. Nilsson P. H.. (2019). The role of complement in liver injury, regeneration, and transplantation. Hepatology 70, 725–736. 10.1002/hep.30508 30653682 PMC6771474

[B32] Toyoda H. Kumada T. Tada T. Sone Y. Kaneoka Y. Maeda A. (2015). Tumor markers for hepatocellular carcinoma: simple and significant predictors of outcome in patients with HCC. Liver Cancer 4, 126–136. 10.1159/000367735 26020034 PMC4439793

[B33] Wu L. J. Y. Ye C. H. Yao Q. C. Li Q. Q. Zhang C. Y. Li Y. D. (2024). The role of serum acylcarnitine profiling for the detection of multiple solid tumors in humans. Heliyon 10:e23867. 10.1016/j.heliyon.2023.e23867 38205321 PMC10776988

[B34] Wu X. N. Xue F. Zhang N. Zhang W. Hou J. J. Lv Y.. (2024). Global burden of liver cirrhosis and other chronic liver diseases caused by specific etiologies from 1990 to 2019. BMC Public Health 24:363. 10.1186/s12889-024-17948-6 38310221 PMC10837876

[B35] Xie D. Zhang G. C. Ma Y. A. Wu D. Y. Jiang S. Zhou S. K.. (2022). Circulating metabolic markers related to the diagnosis of hepatocellular carcinoma. J. Oncol. 2022:7840606. 10.1155/2022/7840606 36532884 PMC9757943

[B36] Yang L. Zhen L. Li Z. Zhu S. Xu W. Luo Q.. (2022). Human liver tissue transcriptomics revealed immunometabolic disturbances and related biomarkers in hepatitis B virus-related acute-on-chronic liver failure. Front. Microbiol. 13:1080484. 10.3389/fmicb.2022.1080484 36532504 PMC9752073

[B37] Yin Y. W. Jin H. J. Zhao W. J. Gao B. X. Fang J. G. Wei J. M.. (2015). The histone acetyltransferase GCN5 expression is elevated and regulated by c-Myc and E2F1 transcription factors in human colon cancer. Gene Expr. 16, 187–196. 10.3727/105221615X14399878166230 26637399 PMC5584536

[B38] Zhao C. H. Li Y. T. Qiu W. He F. X. Zhang W. M. Zhao D.. (2018). C5a induces A549 cell proliferation of non-small cell lung cancer via GDF15 gene activation mediated by GCN5-dependent KLF5 acetylation. Oncogene 37, 4821–4837. 10.1038/s41388-018-0298-9 29773900 PMC6117268

