# Supplementary material for: Transcriptomic and metabolomic analyses reveal the spatial role of carnitine metabolism in the progression of hepatitis B virus cirrhosis to hepatocellular carcinoma
Source: Front Microbiol. 2024 Dec 13;15:1461456. doi: 10.3389/fmicb.2024.1461456 (PMC11671487; doi:10.3389/fmicb.2024.1461456)
Supplement: Supplementary file 1 [file Data_Sheet_1.pdf]

Supplementary Material

1 Supplementary Figures

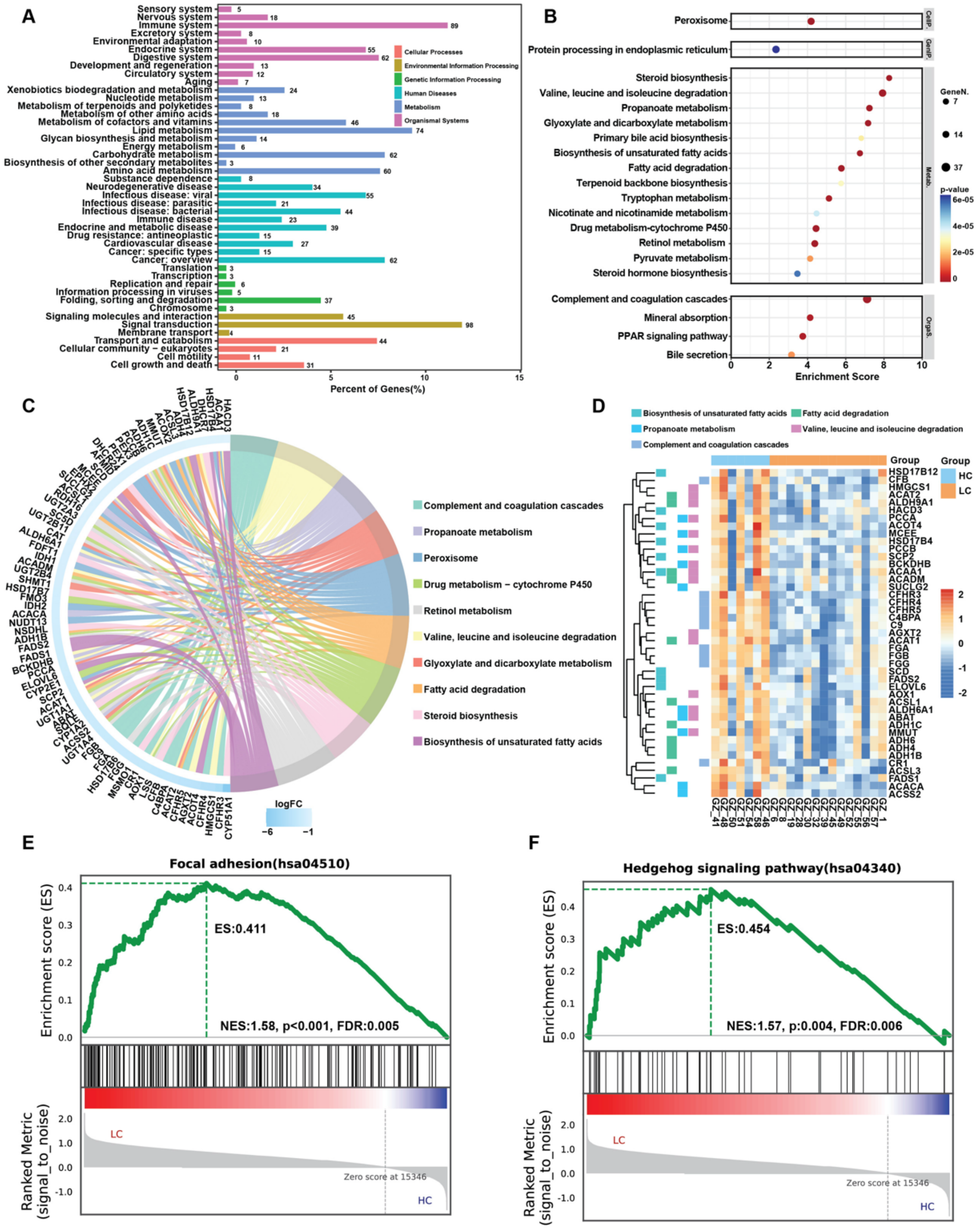

Supplementary Figure 1. Downregulated gene expression in the HBV related liver cirrhosis. A) KEGG pathway classification of downregulated genes in liver cirrhosis. B) Top 20 KEGG enrichment items for downregulated genes in liver cirrhosis. C) Circos map of top downregulated genes and related KEGG pathways. D) Heatmap of selected downregulated DEGs and key related pathways. E) Gene set enrichment analysis (GSEA) comparing genes in patients with liver cirrhosis and healthy controls.

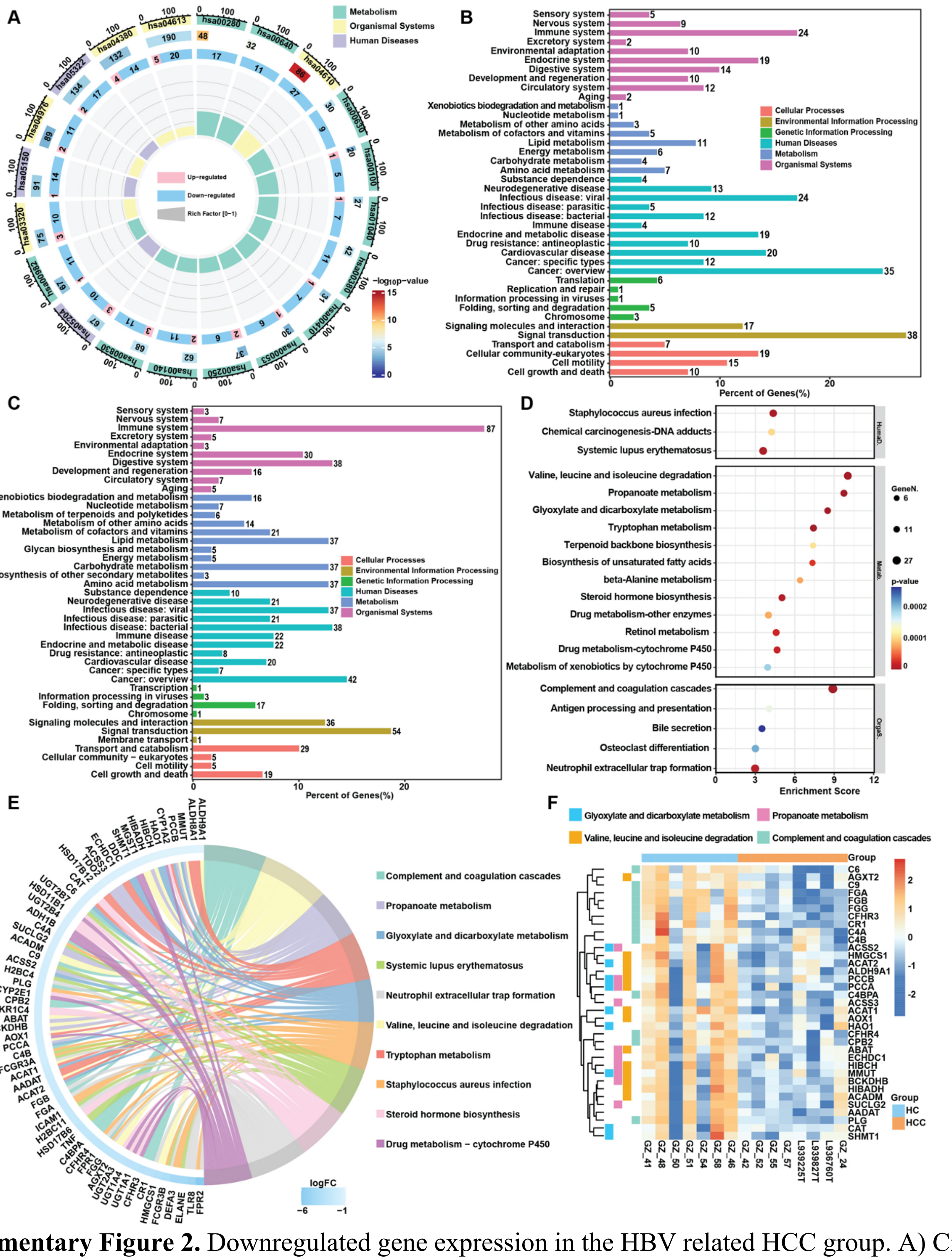

Supplementary Figure 2. Downregulated gene expression in the HBV related HCC group. A) Circos map of DEGs. The circles from inside to outside represent the enrichment factor, the ratio of up- and downregulated genes, background genes, p-value and KEGG level 1 classification. B) KEGG pathway classification of upregulated genes in liver cirrhosis. C) KEGG pathway classification of downregulated genes in the HCC group. D) The top 20 KEGG enrichment terms for downregulated genes in the HCC group. E) Circos plot of the top downregulated genes and related KEGG pathways. F) Heatmap of selected downregulated DEGs and key related pathways.

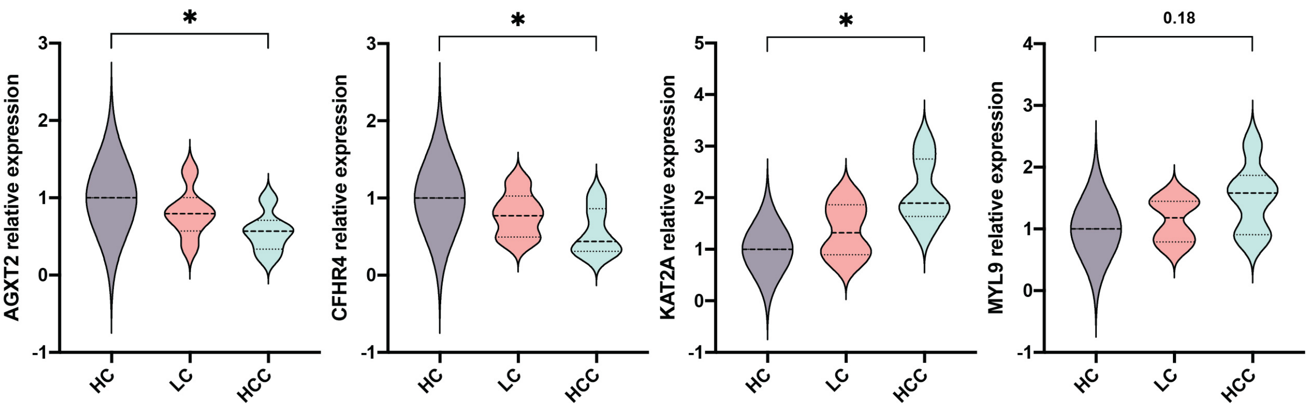

Supplementary Figure 3. q-PCR validation of up-and downregulated genes.

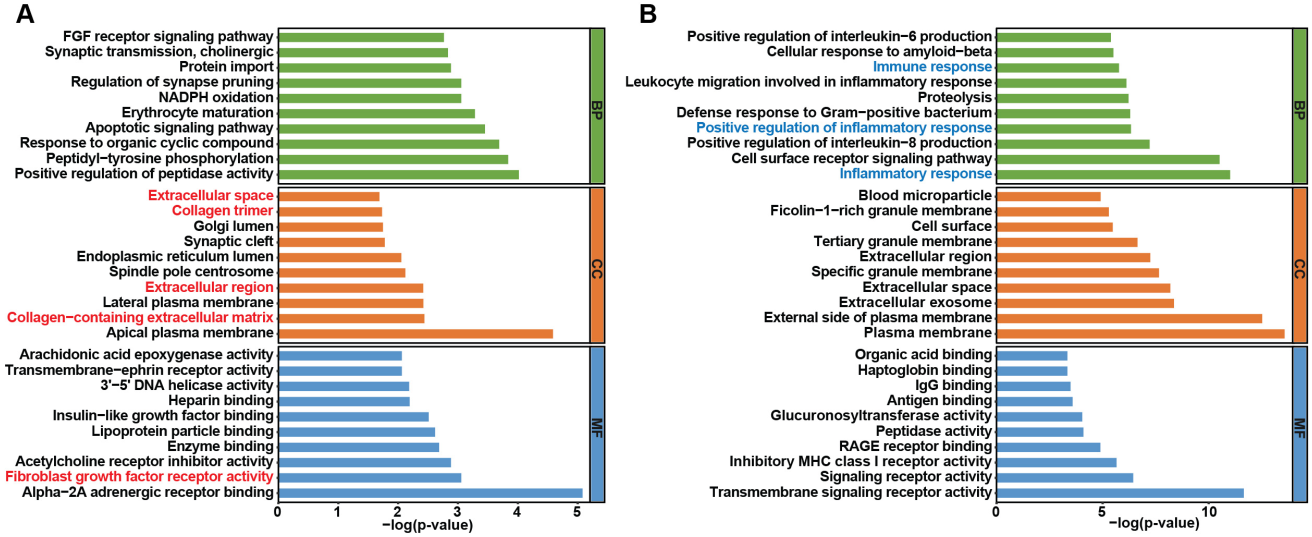

Supplementary Figure 4. GO enrichment analysis of key pathways involved in disease progression. A-B) GO enrichment pathways for genes with increasing (A) and decreasing (B) trends.

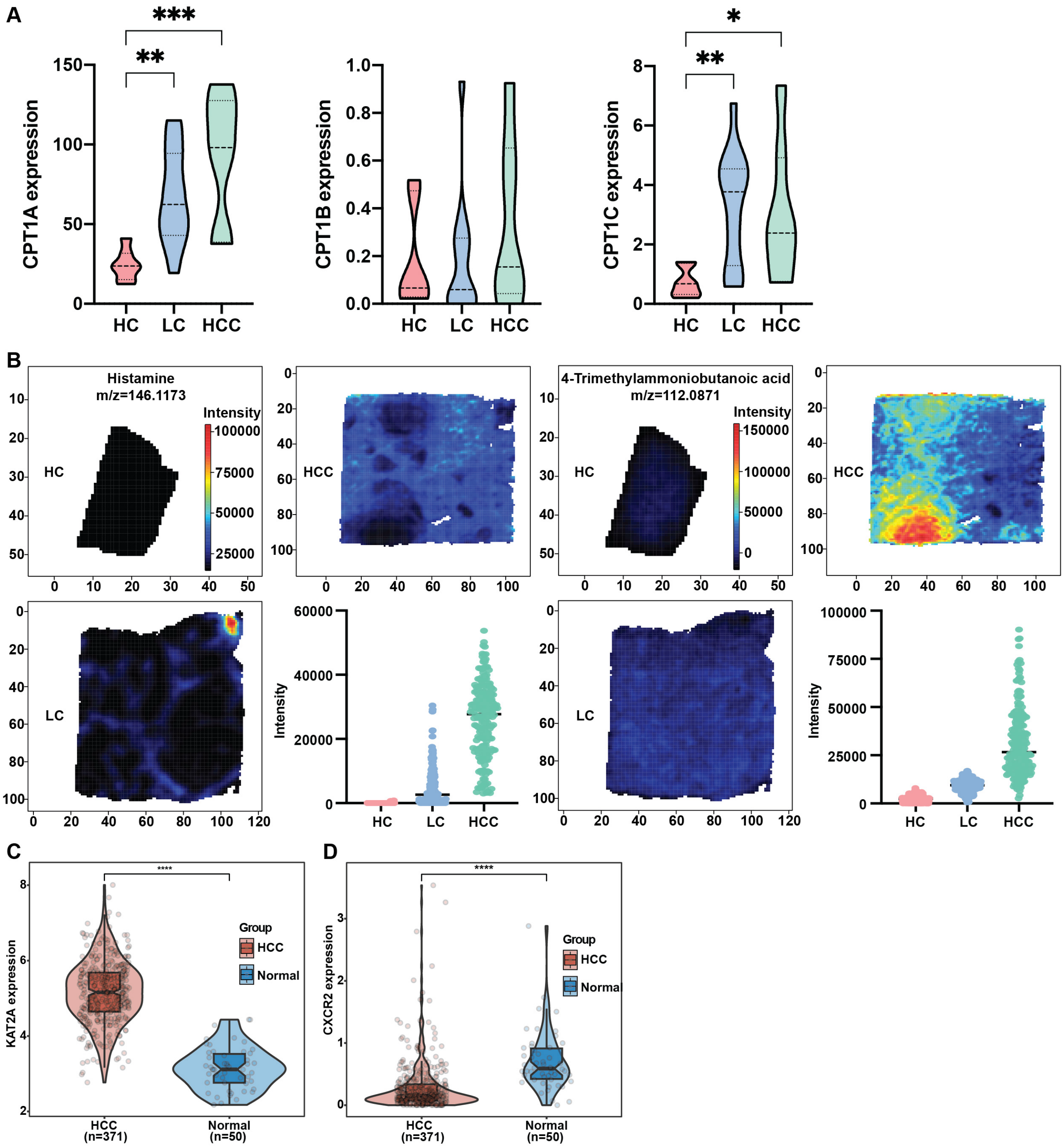

Supplementary Figure 5. Spatial distribution of metabolites and gene expression in the TCGA database. A) Expression of genes regulating carnitine metabolism during disease progression. B) Spatial distribution of metabolites with increasing trends. C-D) The increasing (C) and decreasing (D) trends in gene expression from the TCGA database.

Table S1. Primers sequence of target genes

**Genes**	**Forward primers**	**Reverse primers**
AGXT2	TCCCGGACATCAGTAACCAAG	ACTGGTATCTTTCAGGCATGAAG
CFHR4	CGCGTAGACCATACTTTCCA	ACCCATCTTGTGTGCAGTGA
GAPDH	AATCCCATCACCATCTTCCA	TGGACTCCACGACGTACTCA
KAT2A	TTCCGAGTGGAGAAGGACA	AGCATGGACAGGAATTTGG
MYL9	GCCACATCCAATGTCTTCGC	GCGTTGCGAATCACATCCTC

Table S2. Liver tissue samples Couinaud classification

Sample	GZ_1	GZ_6	GZ_8	GZ_19	GZ_28	GZ_30	GZ_32	GZ_39
Zone	VIII	VIII	V	VIII	VII	V	V	VIII
Group	LC	LC	LC	LC	LC	LC	LC	LC
Sample	GZ_43	GZ_45	GZ_49	GZ_52	GZ_55	GZ_56	GZ_57	
Zone	II	IVa	NA	III	VI	VIII	NA	
Group	LC	LC	LC	LC	LC	LC	LC	
Sample	GZ_24	GZ_42	GZ_52C	GZ_55C	GZ_57C	L93676T	L93922T	L93982T
Zone	V	II	VIII	VIII	IVb	V	VIII	III
Group	HCC	HCC	HCC	HCC	HCC	HCC	HCC	HCC
